# Outcomes and Costs of Poisoned Patients Admitted to an Adult Emergency Department of a Spanish Tertiary Hospital: Evaluation through a Toxicovigilance Program

**DOI:** 10.1371/journal.pone.0152876

**Published:** 2016-04-21

**Authors:** Raúl Muñoz, Alberto M. Borobia, Manuel Quintana, Ana Martínez, Elena Ramírez, Mario Muñoz, Jesús Frías, Antonio J. Carcas

**Affiliations:** 1 Clinical Pharmacology Deparment, Hospital Universitario La Paz, IdiPaz, School of Medicine, Universidad Autónoma de Madrid, Madrid, Spain; 2 Accident and Emergency Department, Critical Care Department, Hospital Universitario La Paz, School of Medicine, IdiPaz, Universidad Autónoma de Madrid, Madrid, Spain; 3 Clinical Toxicology Unit, Hospital Universitario La Paz, Madrid, Spain; National Cheng Kung University, TAIWAN

## Abstract

**Abstract:**

Toxicovigilance is the active process of identifying and evaluating the toxic risks existing in a community, and evaluating the measures taken to reduce or eliminate them.

**Objective:**

Through a validated toxicovigilance program (SAT-HULP) we examined the characteristics of acute poisoning cases (APC) attended in the Emergency Department (ED) of La Paz Hospital (Madrid, Spain) and assessed their economic impact on the health system.

**Material and Methods:**

The active poisoning surveillance system performs a daily search for cases in the hospital´s computerized case records. Found cases are entered into a database for recording of type of poisoning episode, reasons for exposure, causative agent, signs and symptoms and treatment. We carried out a cross-sectional epidemiological study with analytical projection, based on an impact study on cost per survivor. The data for the costs attributable to cases of APC observed at HULP (outpatients and inpatients) was obtained from the based on the information provided by the diagnosis-related groups (DRG) through the corresponding hospital discharge reports (available through SAT-HULP).

**Results:**

During the first 30 month of SAT-HULP operation we found a total of 3,195 APC, a cumulative incidence rate of 1.75% of patients attended in the ED. The mean (SD) patient age was 40.9 (17.8) years and 51.2% were men. Drug abuse accounted for 47.5% of the cases. Suicide attempt was the second most frequent category (38.1%) and other causes accounted for 14.5% of APC. The total cost of hospital care for our hospital rose to €1,825,263.24 (approximately €730,105.30/year) resulting in a permanent occupation of 4 beds/year.

**Conclusions:**

SAT-HULP constitutes a validated toxicovigilance tool, which continuously integrates available data in real-time and helps health services manage APC data flexibly, including the consumption of resources from the health system.

## Introduction

Toxicovigilance is the active process of identifying and evaluating the toxic risks existing in a community, and evaluating the measures taken to reduce or eliminate them and it is one of the basic tasks of the services involved in caring for patients with poisonings [1–7]. This activity requires the necessary data sources, traditionally the programs for reporting the individual (or grouped) cases as well as retrospective and prospective epidemiological studies and surveys [5–11]. In the setting of acute poisoning cases (APC), certain healthcare departments (emergency departments, critical care units) are directly involved in this task [5, 6, 8, 11–13].

Added to the health costs of APC, there is a considerable financial impact on the healthcare system due to the use of resources and the derivative costs of the various healthcare departments involved in caring for patients with poisonings [14, 15].

In December 2010, University Hospital La Paz (HULP) launched the first Clinical Toxicology Unit (CTU) of the Community of Madrid [6]. The CTU’s initial activities included installing a routine active toxicovigilance system through an automated case detection system based on the digitized clinical reports (SAT-HULP) of patients treated in the Emergency Department of the General Hospital (HED), in other words, adults and adolescents older than 14 years of age. This tool has been properly evaluated and validated [6]. This active poisoning surveillance system performs a daily search for cases in the hospital´s computerized case records.

In this study, our objectives were 1) to describe the cases of APC detected by SAT-HULP in the first 2.5 years of operation, calculating the incidence, mortality and readmission, as well as the factors that influence APC; 2) to calculate the impact of APC on the healthcare costs of the Spanish National Health System (NHS) at the regional and national level.

## Material and Method

Data obtained from this activity are kept in an anonymized registry and reported to the Spanish System of Toxicovigilance as required (Sistema de Toxicovigilancia, http://www.fetoc.es/toxicovigilancia/toxicovigilancia.html). This registry is kept within the hospital information system and also in compliance with the Spanish Personal Data Protection Law. Verbal consultation with the Ethical Committee (EC) of our Hospital (Comité Ético de Investigación Clínica del Hospital Universitario La Paz) indicated that no previous patient informed consent or review by the EC would be needed.

A descriptive, cross-sectional epidemiological study was conducted with analytical projection for the entire population treated in the HED during the first 2.5 years of operation of the SAT-HULP program (April 1, 2011 to October 31, 2013).

A descriptive statistical study was performed on the variables. The quantitative variables are expressed as means, standard deviation and range. The qualitative variables are expressed as absolute and relative frequencies. This descriptive statistical study was supplemented by a trend analysis by day of the week using a time series, seasonally adjusted through its breakdown in moving averages, thereby obtaining the corresponding seasonal factors (SF).

### Factors associated with ICU admission and/or mortality and the length of stay

We performed an initial examination of our data using a bivariate analysis, calculating the corresponding raw Odds Ratios of the risk factors related to the combined variable “admission to the intensive care unit and/or mortality”. The independent variables evaluated were the age in groups, sex, location of the initial healthcare act, the type of poisoning, the substance involved, the categories of the Charlson index and the starting consciousness level (defined by the Glasgow scale score). We also included the interaction between the type of poisoning and the type of substance in the model. We subsequently adjusted a logistic regression model using the step-backwards method, with the variables that in the univariate analysis achieved a statistical association (p<0.10) or had a clear clinical and/or biological significance, using the likelihood ratio test to include or exclude the variables that significantly contributed to the model.

Similarly, we conducted the multivariate analysis of the length of stay adjusting for a linear regression model, where we assessed the same predictors including sex. Given that the time variable had a non-normal behavior, we performed a logarithmic transformation of the variable. We ultimately achieved a normal probability Q-Q plot to thereby have a qualitative observation of the degree of approximation to the normal of the residuals.

We considered statistical significance when the p value was <0.05.

### Cost Analysis

The unit of analysis for studying the impact in costs was the patient with APC treated in the HED, identified through the SAT-HULP program and who stayed more than 6 hours either in the HED observation rooms or the “observation without admittance” (OWA) unit and/or who were hospitalized. HED stays longer than 6 hours were counted as a day. The data for the costs attributable to cases of APC observed at HULP was obtained from the repository of the Information System of the General Subdirectorate of the Basic Portfolio of Services of the NHS and the Cohesion Fund of the Ministry of Health, Social Services and Equality (MHSSE) based on the information provided by the diagnosis-related groups (DRG) [16]. The DRG is a patient classification system widely used in Spanish hospitals and in other neighboring countries. The system helps us determine the hospital caseload (type of patients) and is of considerable usefulness in managing and funding hospitals. The information needed to classify each patient in their DRG was found in the corresponding hospital discharge reports (available through SAT-HULP) [6]. Furthermore, we were able to perform an approximation of the costs for patients treated for APC who stayed less than 6 hours in the HED or who were do not admitted to the OWA or to the hospital ward under the classification “emergency, not admitted”, which is equivalent to €122 per episode, as reflected in Order 629/2009 of the Ministry of Health of August 31, which sets the public prices for providing services and activities of a healthcare nature of the network of centers of the Community of Madrid (CAM) [17].

For the comorbidity analysis, we chose the Charlson Index because it has the most advantages and ease of handling in the most common databases. We used the most common adapted version, thereby considering 4 separate groups (0.1–2, 3–4, and >4) [18, 19]. This index was accompanied by the corresponding DRG assigned during the discharge of hospitalized patients and those admitted to the OWA.

The costs for each process were calculated according to the following formula: [equivalent in euros of the weight of the corresponding DRG] * [number of patients classified in this DRG] = total cost in euros. We calculated the costs per survivor for the various age groups and the costs per survivor without readmission. The use of each CAM hospital’s resources attributable to these patients was calculated using the analysis of the mean stay (MS) using the various indicators (proxy) stratified by DRG. This calculation allowed us to compare the complexity and operation of our center in providing care for APC with the standard of SERMAS (Madrid Health Service) and with the other hospitals of our community’s public health system. The data for this analysis was obtained from the 2010 minimum basic data set published by the Ministry of Health, which served as a reference for the first year of the study period [20]. The indicators employed in the study are shown in Table 1.

**Table 1:** Indicators Employed for Evaluating the Use of Hospital Resources.

*Activity*	Indicator	Formula	Description
**Resource consumption**	MSAF	MS/standard MS	Mean stay adjusted for the function of the standard: That which they would have had if they functioned as the standard (of SERMAS)
**Measurement of complexity**	CI	MSAF/SMS (standard MS)	Index of complexity. Less than or greater than 1. Caseload of hospitals that are more or less complex than the standard
	DDC	MSAF-SMS.	Difference in complexity. Difference between the hospital’s MS and the standard MS due to the fact that the hospital’s caseload is more or less complex
**Measuring function**	AMSI	MS of each hospital/MSAF	Adjusted mean stay index. Less than or greater than 1. Operating more or less efficiently than the standard. Helps analyze who can address the same caseload in the fewest number of hospital days
**Difference in function**	DDF	MS of each hospital/MSAF	Difference between the hospital’s MS and the standard MS due to the fact that the hospital’s operation is more or less complex than the standard
**Impact on stays**	IOS	(Difference in MS)*(Discharges by each hospital)	Helps determine:
			• Stays saved (when negative): Stays that each hospital is saving for each DRG in relation to the standard in SERMAS*
			• Avoidable stays (when positive): Stays that each hospital could save for each DRG in relation to the standard in SERMAS

*SERMAS: Servicio Madrileño de Salud (Madrid Health Service)

As an approximation of the clinical results, we determined for each DRG the mortality, the HED attendance and readmissions, setting a time horizon of 30 days for visits for the same cause or one related to the initial diagnosis and of 1 year for hospital readmissions with the same DRG [18].

For the statistical analysis, we used the statistical suite SPSS for Windows version 17.0 and Stata 10 (StataCorp, College Station Text).

## Results

During the period from April 1, 2011 (starting date of the SAT-HULP program) to October 31, 2013 (30 months), the HED of HULP treated a total of 182,502 patients, 3195 of whom were identified as cases of APC (1.75%) using the SAT-HULP system. Considering that HULP serves a population of 752,006 individuals, the rate of APCs treated in the HED in HULP’s reference area is 143/100,000 inhabitants-year.

Table 2 provides a complete description of the patients’ characteristics and the poisoning profile. The mean age of the series was 41 years (SD, 17.9), 31.7% of the patients were younger than 31 years, and there was a slight predominance of males (51.2%). There was a significant percentage (47.2%) of patients with previous psychiatric disease, and 36.8% had a history of alcoholism or addiction (20.6% alcohol, 4.2% cocaine, 2% opioids and 1.8% cannabis).

**Table 2:** Patients Characteristics by Type of Poisoning.

	Suicide (n = 1216)	Abuse (n = 1517)	Accidents (n = 447)	Homicide (n = 15)	OVERALL* (n = 3195)
**Male sex, n (%)**	414 (34.0)	1014 (66.8)	200 (44.7)	8 (53.3)	1636 (51.2)
**Mean age, y (SD)**	40.1 (14.7)	36.8 (15.3)	57.3 (23.3)	31.5 (12.5)	40.9 (17.8)
**History of addiction, n (%) SI**	475 (39.1)	652 (43.0)	43 (9.6)	4 (26.7)	1174 (36.8)
**Psychiatric history, n (%) SI**	955 (78.5)	488 (32.2)	64 (14.3)	1 (6.7)	1508 (47.2)
**Type of substance, n (%)**					
** Pharmaceutical agent**	994 (81.7)	85 (5.6)	233 (52.1)	1 (6.7)	1313 (41.1)
** Drug of Abuse**	194 (16.0)	1420 (93.4)	33 (7.4)	7 (46.7)	1654 (51.7)
** Domestic product**	20 (1.6)	-	56 (12.5)	1 (6.7)	77 (2.4)
** Industrial product**	4 (0.3)	1 (0.1)	13 (2.9)	-	18 (0.6)
** Other (including food poisoning)**	4 (0.3)	11 (0.7)	112 (25.1)	6 (40.0)	133 (4.2)
**Symptoms at admission, n (%) SI**	828 (68.1)	1461 (96.3)	312 (69.8)	14 (93.3)	2615 (81.8)
**Laboratory measurements, n (%) SI**	429 (35.3)	277 (18.3)	223 (49.9)	12 (80.0)	941 (29.5)
**Digestive decontamination, n (%) SI**	504 (41.4)	25 (1.6)	12 (2.7)	2 (13.3)	543 (17.0)
**Use of antidote, n (%) SI**	266 (21.9)	53 (3.5)	106 (23.7)	2 (13.3)	427 (13.4)
**Patient destination, n (%)**					
** Emergency Department Discharge**	957 (78.7)	1427 (94.1)	419 (93.7)	12 (80.0)	2815 (88.1)
** ICU Admission**	26 (2.1)	4 (0.3)	-	-	30 (1.0)
** Ward Admission**	30 (2.5)	9 (0.5)	11 (2.5)	-	50 (1.5)
** Exitus**	1 (0.1)	-	3 (0.7)	-	4 (0.1)
** Transfer**	115 (9.5)	19 (1.3)	6 (1.3)	1 (6.7)	141 (4.4)
** Voluntary discharge**	12 (1.0)	45 (3.0)	5 (1.1)	2 (13.3)	64 (2.0)
** Admission to Psychiatry Unit**	75 (6.2)	13 (0.9)	3 (0.7)	-	91 (2.8)
**Comorbidities, n (%) (according to Charlson Index for hospitalized patients)**					
** Charlson Index 0**	160 (14.4)	20 (3.4)	10 (6.1)	1 (14.3)	191 (10.2)
** Charlson Index 1–2**	109 (9.8)	26 (4.4)	10 (6.1)	-	145 (7.7)
** Charlson Index 3–4**	11 (0.5)	3 (0.5)	6 (3.7)	-	20 (1.1)
** Charlson Index >4**	5 (0.5)	1 (0.2)	1 (0.6)	-	7 (0.4)

*The between groups observed differences are statistically significant (p <0.05) in all cases.

Abuse and/or recreational APCs were the most common (47.5%), followed by suicidal intent (38.1%) and accidents (14.0%). The main substances involved in the APCs were drugs of abuse (51.7% of cases); within these, alcohol was predominant (86.5%). The second most common substances were pharmaceutical agents (41.1%), with benzodiazepines the most common (57.3% of APCs by pharmaceutical agents), followed by oral anticoagulants (9.7%), selective serotonin reuptake inhibitor antidepressants (5.9%), neuroleptics and new anticonvulsant agents (both 4.3%), nonsteroidal anti-inflammatory analgesics (3.1%), paracetamol (3.7%) and cyclic antidepressants (1.6%). In the “other substances” group (n = 133; 4.2% of the total), the most significant APCs were related to food poisoning (38.0%). Among domestic and industrial products (n = 96), caustic agents (20.9%) were almost on par with carbon monoxide (20.5%).

**Fig 1:**
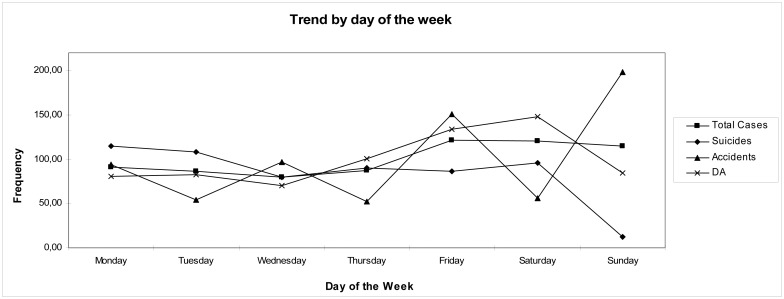
The trend by day of the week. It can be seen as cases of poisoning abusive rises on Friday and Saturday, the suicide type throughout the weekend and accidentally nature clearly in Sunday (likely by the weight of domestic accidents).

We can see from the seasonal factors (Fig 1) that Friday, Saturday and Sunday had higher values for all cases (SF of 121.2%, 120.6% and 114.6% respectively).

**Fig 2:**
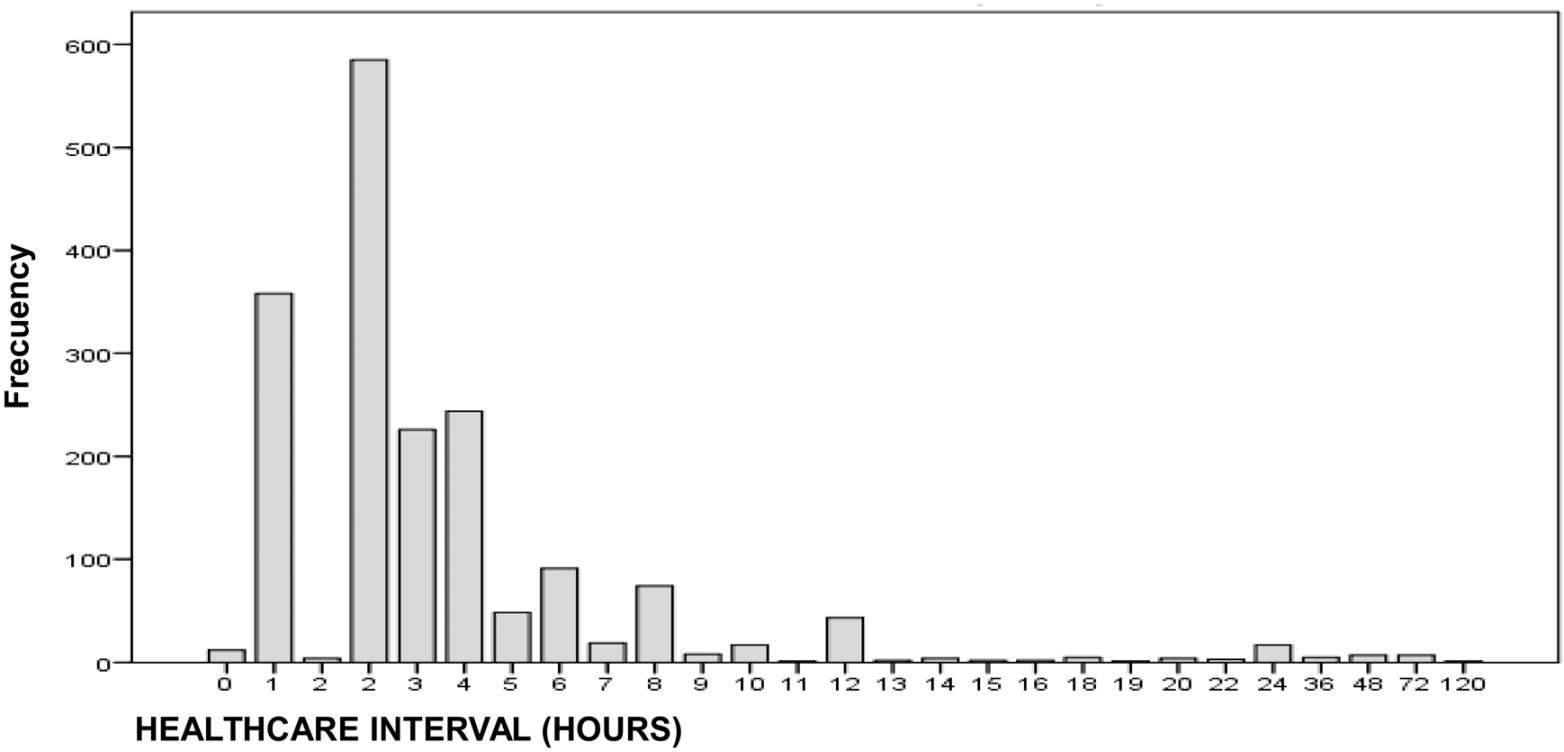
Healthcare Interval. The distribution of time from reported ingestion to presentation by plotting the number of patients presenting in each hour block.

The mean time from the occurrence of the APC to the initial care in the HED in those cases that could be recorded (n = 1790; 56.0% of the total) was 4.14 hours (SD, 6.9; IQR, 2; range, 0–120), with a median time of 2 hours (Fig 2). The mean hospital stay was 1.19 days (SD, 1.24; range, 0–30). Some 58.7% of all APC cases required a stay in the emergency department of more than 6 hours (counted as 1 day) or hospitalization, with a mean stay of 1.99 days (SD, 2.55; range, 1–30) and a median of 1 day. Some 10.2% (192) of all such patients were transferred to the OWA room assigned for hospital stays shorter than 48 hours.

### Factors associated with ICU admission and/or mortality and the length of the hospital stay

A total of 27 patients treated for APC required ICU admission and/or died during the admission (0.85%). Table 3 lists their characteristics. The factors independently associated with the need for ICU admission and/or mortality in the univariate analysis are shown in Table 4.

**Table 3:** Characteristics of Patients Requiring Admission to the Intensive Care Unit (ICU) and/or who Died.

	ICU admission N (%)	Exitus N (%)
**Male sex**	12 (40.0)	3 (75.0)
**Age group**		
** 15 years of age or younger**	-	-
** 16–30 years**	10 (33.3)	-
** 31–45 years**	11 (36.7)	2 (50.0)
** 46–60 years**	7 (23.3)	-
** 61–75 years**	2 (6.7)	-
** 76 years of age or older**	-	2 (50.0)
**Psychiatric history [YES]**	21 (70.0)	1 (25.0)
**Type of poisoning**		
** Suicide**	26 (86.7)	1 (25.0)
** Abusive/Recreational**	4 (13.3)	-
** Accidents**	-	3 (75.0)
** Homicide**	-	-
**Type of substance**		
** Pharmaceutical agent**	26 (86.7)	3 (75.0)
** Drug of Abuse**	4 (13.3)	1 (25.0)
** Domestic product**	-	-
** Industrial product**	-	-
** Other**	-	
**Initial care**		
** Emergency Room/Consultation**	8 (26.7)	1 (25.0)
** Resuscitation Box**	7 (23.3)	1 (25.0)
** Outpatient**	15 (50.0)	2 (50.0)
Comorbidity (according to Charlson Index for hospitalized patients)*		
** Charlson Index 0**	20 (66.7)	-
** Charlson Index 1–2**	9 (30.0)	1 (25.0)
** Charlson Index 3–4**	1 (3.3)	1 (25.0)
** Charlson Index >4**	-	1 (50.0)

*The Charlson Index was calculated in only 3 of the 4 death as this calculation can only be made in hospitalized patients. The 4th patient died in the emergency department.

**Table 4:** Factors Associated with Admission to the Intensive Care Unit and/or in-hospital mortality (univariate analysis).

	OR (95% CI)	p-Value
**Male sex**	0.7 (0.4–1.5)	0.396
**Psychiatric history [YES]**	1.6 (0.4–6.6)	0.551
**Type of poisoning**		
** Suicide**	1.0	-
** Abusive/Recreational**	0.2 (0.1–0.4)	0.000
** Accidents**	0.1 (0.0–7.6)	0.006
** Homicide**	0.0	0.567
**Type of substance**		
** Pharmaceutical agent**	1.0	-
** Drug of Abuse**	0.1 (0.0–0.3)	0.000
** Domestic product**	0.0	0.195
** Industrial product**	0.0	0.531
** Other**	0.0	0.089
**Initial care**		
** Emergency Room/Consultation**	1.0	-
** Resuscitation Box**	50.8 (16.6–155.5)	0.000
** Outpatient**	4.3 (1.9–9.6)	0.000
**Comorbidity (according to Charlson Index for hospitalized patients)**		
** Charlson Index 0**	1.0	-
** Charlson Index 1–2**	1.1 (0.6–2.1)	0.713
** Charlson Index 3–4**	8.1 (2.9–22.8)	0.000
** Charlson Index >4**	8.8 (1.8–43.9)	0.001

The significantly associated risk factors include the initial care in the resuscitation box and a Charlson Index >3. The protective factors were APCs from accidental causes and those caused by drugs of abuse. Table 5 shows the results of the multivariate analysis. We can see that the starting level of consciousness was a protective factor related to the severity of the APC (OR, 0.67; range: 0.59–0.76), as well as the type of poisoning (abuse or recreational) (OR, 0.21; range: 0.08–0.56). In contrast, the initial care by prehospital emergency services is shown to be a risk factor, with an OR of 2.80 (range, 1.22–6.45). Adjusting for the type of poisoning and the location of initial care, we see that for every additional point in the Glasgow scale, there was an approximately 30% lower probability of having a case of severe poisoning. Due to the colinearity between the variables, we did not assess the possible interaction between the type of poisoning and the type of substance involved.

**Table 5:** Multivariate Analysis Risk factors for severe poisoning (ICU admission and/or exitus). LR Χ^2^ = 88.97, Prob> Χ^2^ = 0.0000 and PseudoR^2^ = 0.2519.

	Standard Error	Significance (p)	OR	95% CI
**Initial Care Resuscitation Box**	2.795	0.092	3.646	0.811–16.385
**Initial care Outpatient**	1.192	0.015	2.801	1.217–6.448
**Type of poisoning Abuse/Recreational**	0.105	0.002	0.207	0.077–0.558
**Initial level of consciousness (GCS score)**	0.043	0.000	0.668	0.588–0.758

Similar to the previously described epigraph, we conducted the multivariate analysis of the length of the hospital stay adjusting for a linear regression model, where the predictors of this stay (with a p<0.05) would be age, Glasgow scale score and a history or not of psychiatric disorders. The results can be seen in Table 6. We therefore arrived at a predicted MS value of 2.98 days.

**Table 6:** Multivariate Analysis Predictors of mean hospital stay. F = 9.655, p = 0.000, R2 = 0.076, R2 adjusted = 0.068.

	Standardized coefficients	t	Significance	95% CI for B (nonstandardized)	
	Beta			LI	LS
**Constant**	-	5.766	0.000	0.602	0.882
**Initial level of consciousness (GCS score)**	-0.185	-3.612	0.000	-0.044	-0.206
**Age**	0.166	3.249	**0.001**	0.001	0.002
**Psychiatric history [SI]**	-0.119	-2.338	0.020	-0.078	-0.034

### Estimate of the financial impact of APCs

The cost of APCs for patients who remain less than 6 hours in the HED (emergency department without hospitalization) ultimately rose to a total of €345,992 (2836 episodes).

Through the corresponding DRGs, we also established the assessment of the treatment of hospitalized patients, classifying them into clinically similar groups and with similar resource consumption, such as 582, 426, 428, 449 and 750, which were the most common of the series (Table 7). Therefore, the cost of APCs in hospitalized patients (calculated using all DRGs) ultimately rose to €1,479,271.24. Table 8 includes the data on the costs by most common DRGs, where DRGs 582, 426, 428, 449 and 750 represented 0.11%, 0.05%, 0.06%, 0.03% and 0.03%, respectively, of the public healthcare expenditure in hospital and specialist services of the autonomous communities. The least common DRGs (430, 751, 427 and 450) represented 0.62%, 0.05%, 0.04% and 0.02%, respectively, totaling 1.1% of this expenditure, which in turn represents 54.77% of the total consolidated public healthcare expenditure of the national health system (overall for the country) for the activity of 2011.

**Table 7:** Frequencies of the Various DRGs in Patients Hospitalized for Acute Poisoning in HULP (OWOH and hospitalization).

*DRG*	*DESCRIPTION*	*Weight*	*Unit Cost*	*Frequency*, *%*
**428**	Personality disorders and impulse control	0.7208	€3,546.04	37.0
**582**	Injuries, poisonings and toxic effects of drugs, except for multiple trauma with major complications	1.4270	€7,020.34	11.7
**426**	Depressive neurosis	0.9358	€4,603.97	11.4
**750**	Alcohol abuse or dependence with complications	0.9408	€4,628.55	8.6
**449**	Poisoning and toxic effect of drugs. Older than 17 years with complications	0.6690	€3,291.39	6.4
**430**	Psychosis	1.3410	€6,597.63	4.7
**427**	Neurosis, except for depression	0.8194	€4,031.21	4.5
**751**	Alcohol abuse or dependence without complications	0.8128	€3,999.03	3.9
**450**	Poisoning and toxic effect of drugs. Older than 17 years without complications	0.4656	€2,290.47	3.3
**Other**	Miscellaneous (84,102,127,395,425,429,432,455,544,744,745)	See Reference 43:		8.5

**Table 8:** Total Costs and Costs by Survivor without Hospitalization of the Main DRGs for the Various Age Intervals.

	DRG 428			DRG 582			DRG 426			DRG 750			DRG 449		
*Age groups*, *y*	*Cases*	*Costs*	*Cost by Survivor without Readmission*	*Cases*	*Costs*	*Cost by Survivor without Readmission*	*Cases*	*Costs*	*Cost by Survivor without Readmission*	*Cases*	*Costs*	*Cost by Survivor without Readmission*	*Cases*	*Costs*	*Cost by Survivor without Readmission*
**Younger than 15**	2	€7,092.08	**€7,092.08**	0	€0	**€0**	0	€0		0	€0	**€0**	0	€0	**€0**
**15–30**	26	€92,197.04	**€3,841.54**	10	€70,203.4	**€7,020.34**	5	€23,019.85	**€4,603.97**	3	€13,885.65	**€4,628.55**	5	€16,456.95	**€3,291.39**
**31–45**	57	€202,124.28	**€3,743.04**	15	€105,305.1	**€9,573.19**	18	€82,871.46	**€6,905.95**	17	€78,685.35	**€7,868.54**	6	€19,748.34	**€3,291.39**
**46–60**	34	€120,565.36	**€3,653.49**	8	€56,162.72	**€9,360.45**	14	€64,455.58	**€5,859.60**	10	€46,285.5	**€5,142.83**	6	€19,748.34	**€3,291.39**
**61–75**	13	€46,098.52	**€3,841.54**	5	€35,101.7	**€8,775.43**	2	€9,207.94	**€4,603.97**	0	€0	**€0**	3	€9,874.17	**€3,291.39**
**Older than 75**	1	€3,546.04	**€3,546.04**	4	€28,081.36	**€7,020.34**	2	€9,207.94	**€4,603.97**	0	€0	**€0**	3	€9,874.17	**€3,291.39**

Furthermore, Table 8 contains the calculations for the cost per survivor according to DRG by age group (the mortality is so low in almost all cases that the cost matches the unit costs) and the cost to the system of a patient with APC surviving without requiring an acute hospital readmission (perhaps more interesting from the funder’s point of view).

Therefore, the total cost of hospital care for patients with APC detected during the 30 months of the program’s operation rose to €1,825,263.24 (which represents approximately €730,105.30/year).

**Fig 3:**
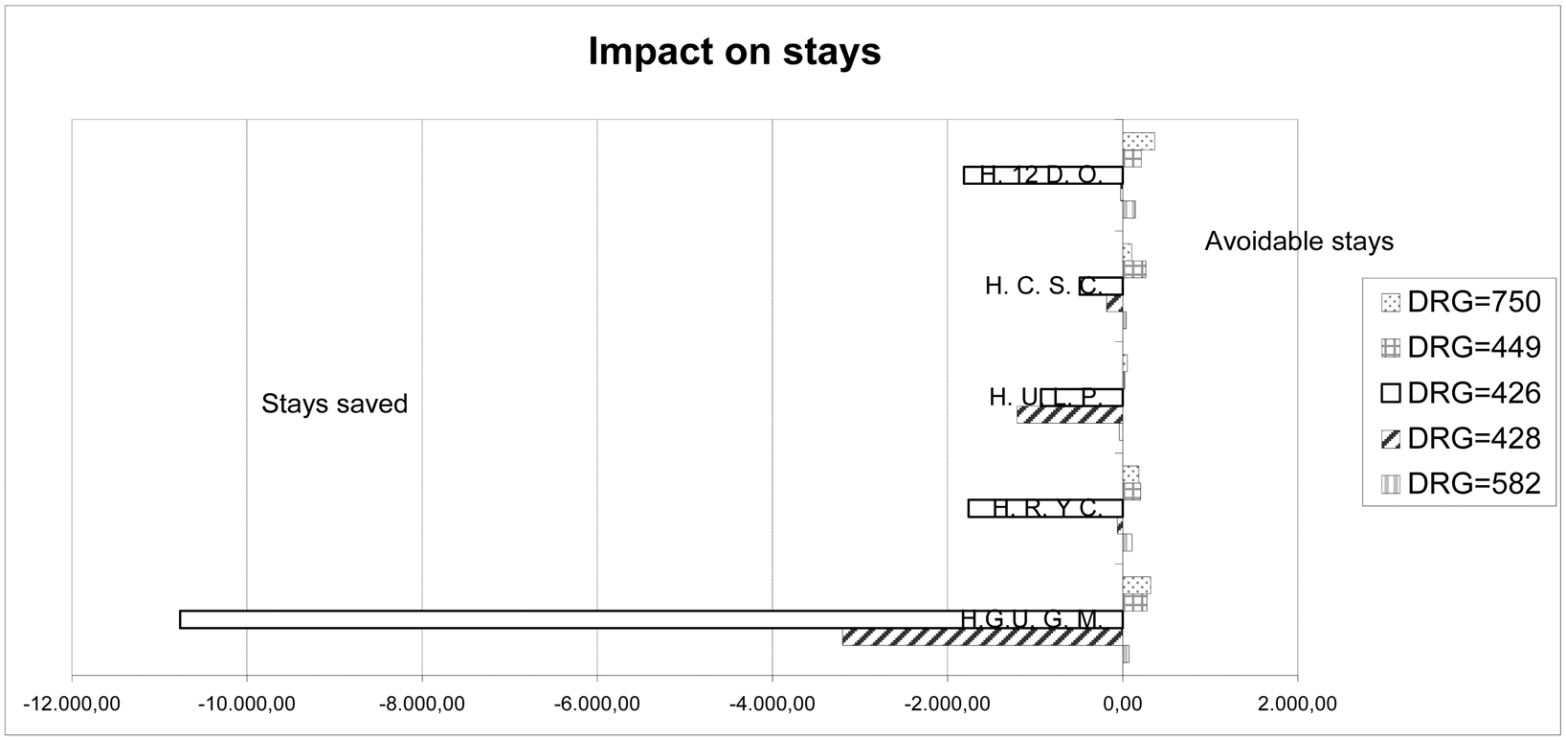
Impact on stays (IOS) among the Main University Hospitals of the Community of Madrid with 1100 Beds or More Beds. This figure represent the number of stays saved (negative figures) or the avoidable stays (positive figures) for each hospital in relation to the standard mean stay in the SERMAS for the most relevant DRG (see Table 6 for description). For details on calculation see also Table 1. H.U.L.P: University Hospital La Paz. H. 12 D. O: Hospital 12 de Octubre. H.C.S.C: Hospital Clínico San Carlos. H.R.Y.C: Hospital Ramón y Cajal. H.G.U.G.M: General University Hospital Gregorio Marañón.

**Fig 4:**
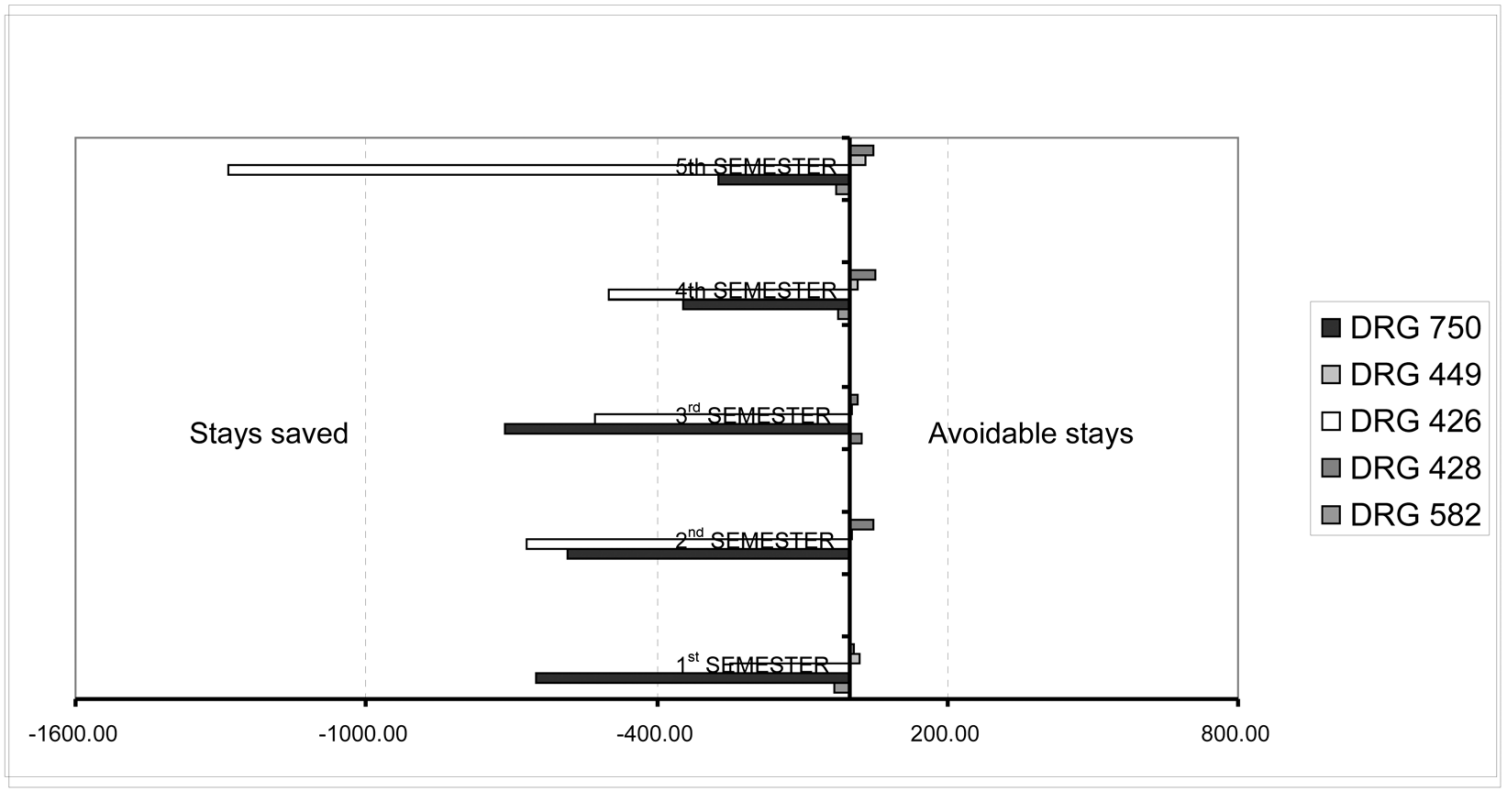
Evolution of IOS in HULP by semester and for each main DRG. The evolution of this indicator is necessary for the appropriate Benchmark.

**Fig 5:**
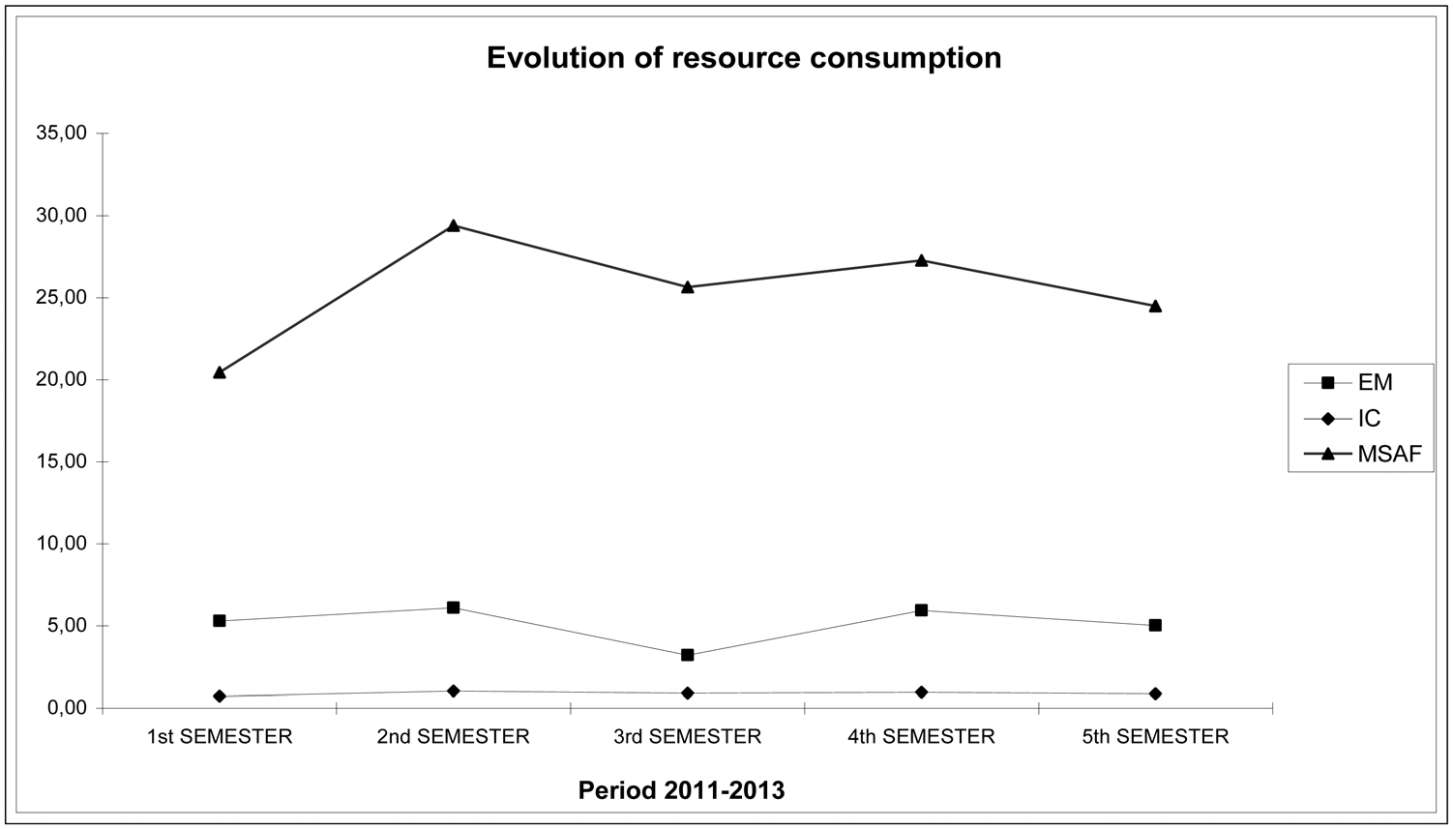
Evolution of quarterly resource consumption (see Table 1). It is seen as long as the complexity of the hospital (CI) is practically constant, the mean stay of our hospital (MS) remains at significantly lower values compared to the standard of all hospitals in the Community of Madrid (MSAF) for all DRGs involved.

**Fig 6:**
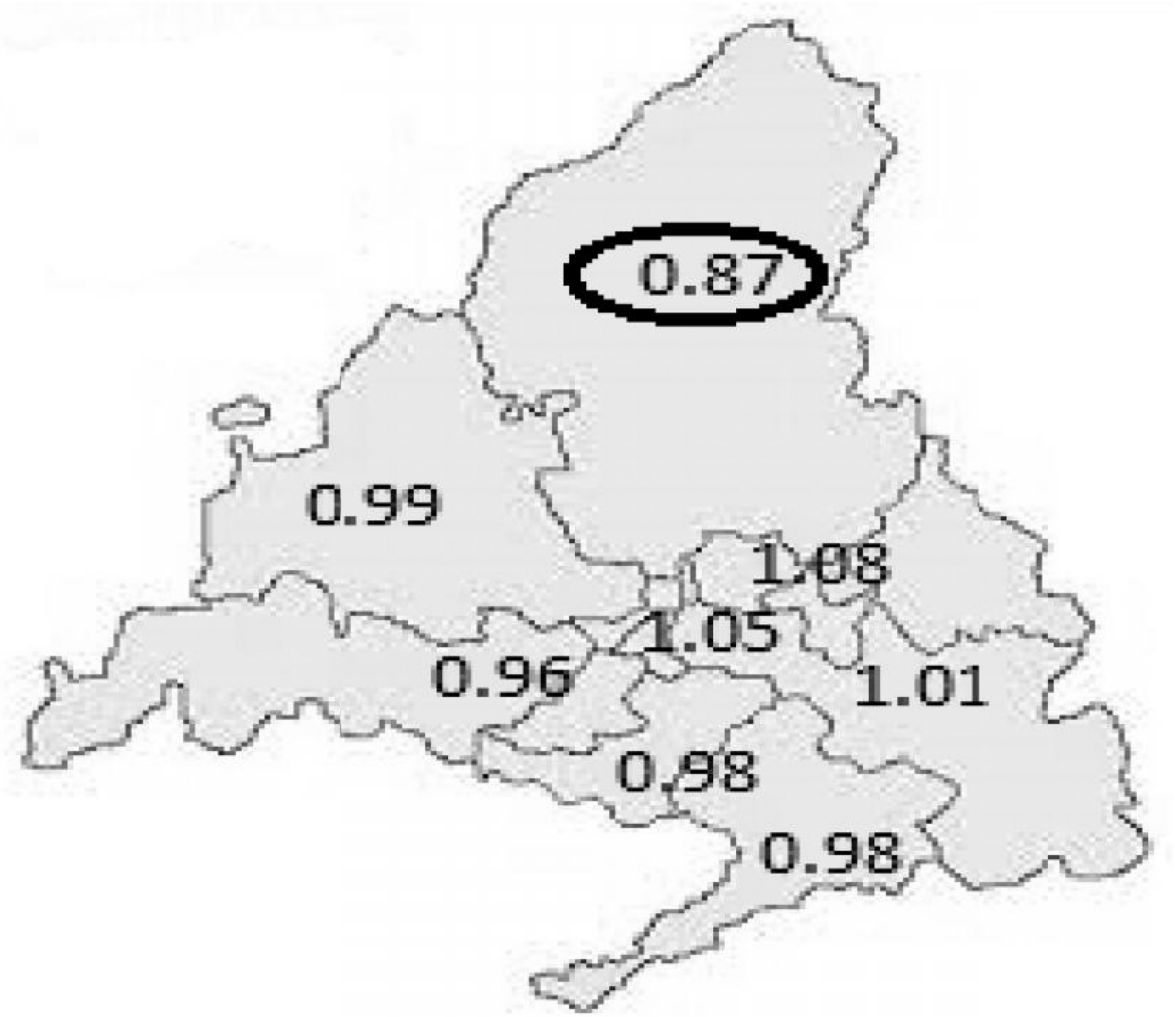
Complexity Index (CI, see Table 1) of the various healthcare areas of the SERMAS. >1, the caseload of the reference hospital is more complex than the regional standard. <1, the caseload of the reference hospital is less complex than the regional standard. The values represented correspond to the higher complexity hospitals. The index for HULP as a reference center appears surrounded by an oval. Reprinted from figure 6 under a CC BY license, with permission from Raul Muñoz, original copyright 2015.

Once the overall indicators for the hospital’s caseload and operation were calculated, we proceeded to individually compare the differences between the mean stay at our center and the standard mean stay of SERMAS (Department Health Service) for each DRG. This procedure helped us compare the operation of HULP (adjusting by caseload) with that of the other centers with similar characteristics in the Community of Madrid, as well as the evolution of our center’s operation by quarter. The results are shown in Figs 3 and 4. Fig 5 shows the evolution of resource consumption for the study period (this is necessary for the Benchmarking of CTU). Fig 6 shows our hospital’s situation compared with that of the other reference centers of the region in terms of the complexity of the caseload (Complexity Index, 0.87), coming in sixth in complexity after the the rest of high complexity hospitals SERMAS (La Princesa, La Paz, Doce de Octubre, Ramon y Cajal, Clínico San Carlos, Gregorio Maranon and Puerta de Hierro) for GRDs referrals. Has to be taken in mind that our hospital does not perform transplants adult (except kidney) and sociodemographic characteristics of the reference population. Can be consulted calculations and data source used in S1–S3 Files.

Some 93.59% of the patients who were hospitalized or admitted to the OWA had a single episode during the follow-up period. HED attendance for APC according to the Charlson Index category (presence of comorbidities) was as follows: 5.8% for a Charlson Index of 0, 8.3% for a Charlson Index of 1 to 2 and 5.0% for a Charlson Index of 3 to 4. There are no cases of attendance for Charlson Indices >4. The cost overrun for the NHS represented by these cases is included in the calculations shown above.

## Discussion

Toxicovigilance, as an essential part of the portfolio of services of a Clinical Toxicology Unit, consists of a healthcare information system that continuously and systematically collects data on the effects of xenobiotics in human health and its impact on healthcare systems [5, 6].

This activity is encompassed within the syndromic surveillance systems of morbidity and mortality. The main objective of these systems is the early detection of events that can significantly affect public health. The systems must undergo frequent updates and provide rapid access to information. The system facilitates the implementation of strategies for the early and quick resolution of detected problems, thus reducing the magnitude of the damage for the population and the burden on the healthcare system [6–8]. An important attribute of the SAT-HULP program is its ability to provide disaggregated data for other variables of interest such as age, sex and comorbidity, thereby providing additional advantages for redistributing healthcare resources among population groups within a scenario of heavy healthcare demand [21]. In addition, this cases capture system represents a significant strength compared to similar studies based on reviews of clinical and administrative databases (coded definitive diagnoses) because these can lose a significant proportion of APC [22, 23].

It is important to note that our program robustly detects that the proportion of men in APC is slightly higher, which corresponds with the studies performed in Spain, and is in contrast to the results of series in other countries, where the female/male proportion can reach a ratio of 3/1 [4, 6, 9, 13, 24–27].

With regard to poisonings from substances of abuse, we can link our series to a pattern of festive consumption, preferentially associated with weekends (similar to the results of published studies) [24, 27–29]. The frequencies of the type of drug used (alcohol 83.1%, cocaine 6.6%, cannabis 2.7%, amphetamines 0.8% and opioids 0.5%) differs slightly from the majority of sources consulted, in which cannabis is ahead of cocaine [14, 29]. Furthermore, 15.7% of the abuse APCs was caused by a combination of alcohol and drugs or by multiple drug use. Some 8.7% of the cases were associated with psychoactive drugs, for the most part benzodiazepines (6.9%), which confirms the increasing progress of mixed poisonings caused by the combination of alcohol, various drugs and psychoactive pharmaceuticals [13, 24, 26, 27, 29, 30].

As with most of the published studies, we can see that acute poisonings from drugs of abuse mainly occur in young individuals, towards whom efforts to prevent this public health problem should be directed. We can see a high percentage (43%) of patients with a history of alcoholism or addiction, and 32.2% have prior psychiatric disease. Unlike other published series, the consumption of multiple drugs was not a risk factor in our study for the increase in ICU admissions [1, 6, 30]. It is worth noting that after the overwhelming media and political impact resulting from the use of ecstasy and amphetamines in the mid-1990s, their usage trends seem to be stable. The use of cannabis, however, has grown considerably, with an annual usage rate of 11.25% among the 15–64 age group and 19.85% for the 15–34 age group. It is estimated that there are more than half a million daily consumers of cannabis in Europe [29, 30]. Thus, the HULP CTU toxicovigilance system can help determine the characteristics and trends of problematic drug use and can act as an early warning system for detecting changes in the phenomenon (new drugs or usage forms) [6, 7].

Our results for the analysis of risk factors related to poisoning severity (ICU admission and/or exitus) are fairly similar to those recorded in the literature. The initial consciousness level, the emergency department’s point of care, the type of APC (abuse or recreational) and care by prehospital emergency services all were shown to be robust predictors (after adjusting for age and sex). There was no interaction between age and sex or the degrees of comorbidity (Charlson Index categories) [30–32]. An important part of our study focused on the analysis of resource consumption and costs for hospitalized patients, where the impact of age on the length of stay deserves a special mention. There are a number of studies that have examined the impact of age on mortality and the length of the hospital stay, but their results have not been conclusive [19,]. In our study, age was a significant predictor of the length of the hospital stay, although its weight in the construction of the model was low. One of the possible explanations for the relatively modest role of age is that young patients who require hospitalization tend to have proportionally more severe acute disease processes and lower physiological scores. As a result, they have comparatively similar mortality rates and hospital stays to those of older patients [19, 31, 33], which would mean that the equation resulting from our hospital stay model should only be used as guidance. If we compare the costs at our center with those of other similar tertiary hospitals within the Madrid Health Service (adjusting for age, initial consciousness level and the presence or absence of psychiatric antecedents), the results of the model help us detect whether there are significantly larger differences in the mean stay, thereby establishing an exploratory overview of the practices and caseload in this center.

Care for APC is a process that consumes a significant amount of resources in terms of specialized personnel in emergency departments and ICUs and requires the participation of other departments (e.g., psychiatry, pharmacy and toxicology), thereby highlighting the importance of Multidisciplinary Functional Units for toxicology care. Despite the efforts of scientific societies and consensus groups, few methods have been developed to manage and assess the appropriate use of resources and the length of stays for APC [14, 15, 22, 23, 34]. In this context, SAT-HULP represents a toxicovigilance tool that also helps integrate the data sources needed to continuously calculate the costs and resource consumption associated with providing care for APC thus complementing traditional systems [14, 22, 23, 35].

Our study reveals significant variability among centers in terms of resource consumption for the various DRGs involved. Despite current debate about this information source [36, 37], the DRG system has the advantage of grouping patients with similar processes, which is comparable among various units and centers. The analysis of the variability in the use of resources helps us understand whether the use of healthcare teams with greater experience in providing care for APC helps achieve better results, which is consistent with other studies that have linked the caseload of hospitals for complicated processes and critically ill patients to better results [36–38]. It is of epidemiological interest and useful for healthcare planning to observe how the evolution of this resource consumption by DRG is subject to seasonal variations, unlike the complexity of the treated patients (which remains steady), a phenomenon already observed in various publications [14, 15, 17, 22, 23, 39].

With regard to the influence of comorbidity (as assessed through the Charlson Index), the underlying hypothesis is that adverse health results increase as comorbidity increases. However, a number of studies that have analyzed long-term readmissions have stated that the influence of comorbidity is determined by the relationship between 2 competitive risks: mortality and readmission. Due to the nature of the Charlson Index, which excludes acute diseases, the index can behave as a protective factor against mortality (as observed in readmission data by Charlson Index), although it is not really in the purely epidemiological sense. This paradoxical effect can be even more apparent in the short-term results of severe processes, due to the fact that hospital mortality can be greater among patients with no or with minimal comorbidity than in other patients (contrary to a priori expectations). This could therefore explain the absence of statistical significance for the various Charlson Index categories, as well as its possible interaction with age as a predictor of mortality [33, 36, 40, 41].

After analyzing readmissions by DRG and in accordance with the data reported by various authors [14, 22, 23, 39, 41–44] we observed that patients who were readmitted had a lower severity of APC and medical comorbidity, with a more favorable clinical outcome but with greater psychiatric comorbidity and an increased risk of attempted suicide. These patients are therefore candidates for assessment and subsequent treatment by the psychiatry unit, both in the acute phase and the medium to long-term, as they would correspond at DRGs 750, 428 and 426. By observing the costs per survivor without readmission, we see that these patients require a significant amount of resources from the system (Table 6b). As a result, the Department of Psychiatry plays a special role in the process of comprehensive care for APCs, thereby highlighting one the strengths of our center’s organizational care: All cases of APC that present as autolytic attempts are evaluated psychiatrically. This policy supports the need for the multidisciplinary nature of the CTU as it has been shown in recent studies [22, 34].

## Conclusions

Finally, our study shows that the costs of APC represent a considerable sum within the total consolidated public health expenditure of the NHS (€32,715,760,000) for the activity corresponding to 2011. The cost per hospitalized patient (€4,120.53) is greater than a number of published results ($1,776 on average) and lower than those attribute to the harmful use of opioid analgesics ($15,884 to $18,388) but very similar to the results for the most complete and recent studies for patients who consume more resources ($4,821.49 in upper quintile). The latter figures are close to our results for survivors without readmission aged 15 to 45 years, who represent 50% of these costs [14,15,22,23,39]. Although these figures should be assessed with caution due to the differences among the various national health systems, we should consider the similarity of the institutions and the healthcare departments involved, which belong to the public healthcare networks in almost all cases. Furthermore, from the perspective of the impact on costs at the regional level and of the opportunity cost of using hospital beds and taking into account that the annual expenditure by bed in specialized care in the Community of Madrid for the same exercise [20, 44] rises to approximately €187,193, we would have about 4 beds in our center (752,006 population attended by our hospital) occupied on a permanent basis by these patients for a year (the ratio of beds/1000 inhabitants in the Community of Madrid is 2.87) [20]. Finally, we can see that for DRGs 582, 426, 428, 449 and 750, the proportion of cases due to APC (out of the total) at 1 year in our hospital is 21.55%, 55.1%, 53%, 34.6% and 41%, respectively. If we roughly extrapolate to the rest of Spain [16, 44], this would represent total costs of €8,029,618.93, €10,695,298.52, €11,111,536.42, €34,139.90 and €3,653,636.81. The figures mentioned above represent a significant impact on costs for the NHS. Therefore, the majority of efforts in preventive strategies and the planning of health services should be directed towards this type of young patient, taking into account their special social relevance [14, 15, 22, 23, 27, 29, 31, 39].

In conclusion, SAT-HULP constitutes a validated toxicovigilance tool, which continuously integrates available data sources in real-time and helps health services manage APC data flexibly. The results from 30 months of operation show APC characteristics similar to those described in the literature. SAT-HULP thereby helps assess the evolution of the severity of APC in our community, including the consumption of resources from the health system, which rises to a mean cost per patient (all inclusive) of €571.29.

## Supporting Information

S1 File Analysis of the length of stay and resources. You can see the calculations of the indicators described in Table 1 for our hospital and other hospitals of the network SERMAS DRGs considered. They are calculated specifically for hospitals also considered high complexity.
(XLS)

S2 File Analysis of the length of stay and resources in the HULP. You can see the calculations of the indicators described in Table 1 for our hospital DRGs considered. It focuses on the consumption of resources of our hospital during the study period.
(XLS)

S3 File Total hospital hospitalization DRGs network SERMAS. The data from all the hospitals in the network are shown.
(XLS)
